# Equine bone marrow-derived mesenchymal stromal cells reduce established *S*. *aureus* and *E*. *coli *biofilm matrix *in vitro*

**DOI:** 10.1371/journal.pone.0312917

**Published:** 2024-10-31

**Authors:** Sarah M. Khatibzadeh, Linda A. Dahlgren, Clayton C. Caswell, William A. Ducker, Stephen R. Werre, Sophie H. Bogers

**Affiliations:** 1 Department of Large Animal Clinical Sciences, Virginia-Maryland College of Veterinary Medicine, Virginia Tech, Blacksburg, VA, United States of America; 2 Department of Biomedical Sciences and Pathobiology, Virginia-Maryland College of Veterinary Medicine, Blacksburg, Virginia, United States of America; 3 Department of Chemical Engineering, College of Engineering, Virginia Tech, Blacksburg, VA, United States of America; 4 Laboratory for Study Design and Statistical Analysis, Virginia-Maryland College of Veterinary Medicine, Blacksburg, Virginia, United States of America; Lady Hardinge Medical College, INDIA

## Abstract

Biofilms reduce antibiotic efficacy and lead to complications and mortality in human and equine patients with orthopedic infections. Equine bone marrow-derived mesenchymal stromal cells (MSC) kill planktonic bacteria and prevent biofilm formation, but their ability to disrupt established orthopedic biofilms is unknown. Our objective was to evaluate the ability of MSC to reduce established *S*. *aureus* or *E*. *coli* biofilms *in vitro*. We hypothesized that MSC would reduce biofilm matrix and colony-forming units (CFU) compared to no treatment and that MSC combined with the antibiotic, amikacin sulfate, would reduce these components more than MSC or amikacin alone. MSC were isolated from 5 adult Thoroughbred horses in antibiotic-free medium. 24-hour *S*. *aureus* or *E*. *coli* biofilms were co-cultured in triplicate for 24 or 48 hours in a transwell plate system: untreated (negative) control, 30 μg/mL amikacin, 1 x 10^6^ passage 3 MSC, and MSC with 30 μg/mL amikacin. Treated biofilms were photographed and biofilm area quantified digitally. Biomass was quantified via crystal violet staining, and CFU quantified following enzymatic digestion. Data were analyzed using mixed model ANOVA with Tukey post-hoc comparisons (p < 0.05). MSC significantly reduced *S*. *aureus* biofilms at both timepoints and *E*. *coli* biofilm area at 48 hours compared to untreated controls. MSC with amikacin significantly reduced *S*. *aureus* biofilms versus amikacin and *E*. *coli* biofilms versus MSC at 48 hours. MSC significantly reduced *S*. *aureus* biomass at both timepoints and reduced *S*. *aureus* CFU at 48 hours versus untreated controls. MSC with amikacin significantly reduced *S*. *aureus* biomass versus amikacin at 24 hours and *S*. *aureus* and *E*. *coli* CFU versus MSC at both timepoints. MSC primarily disrupted the biofilm matrix but performed differently on *S*. *aureus* versus *E*. *coli*. Evaluation of biofilm-MSC interactions, MSC dose, and treatment time are warranted prior to testing *in vivo*.

## Introduction

Biofilms are a common and critical problem in orthopedic infections, such as septic synovitis and implant-associated infections. Infection-associated complications and resistance of biofilms to treatment contribute to increased treatment costs and treatment failure in human [1–3] and veterinary patients [4–7]. A biofilm is a community of bacteria adhered to a surface or to each other and encased in a self-secreted extracellular matrix of carbohydrates, proteins, and DNA [8–14]. Biofilms contribute to antimicrobial resistance by shielding indwelling bacteria from immune cells and antimicrobials [12,15–18], facilitating bacterial metabolic downregulation [19,20], and promoting exchange of antimicrobial resistance genes [7]. Resistance of biofilm-bound bacteria to antimicrobials and immune cells contributes to persistent infection in the face of parenteral and regional antimicrobial administration [6,21–23]. Multi-stage surgical removal of infected implants and debridement of affected tissue are often required in conjunction with prolonged antimicrobial therapy for infection resolution [4,5,24].

Despite aggressive and prolonged medical and surgical therapy, sequalae of orthopedic infections involving biofilms include irreversible tissue damage due to chronic inflammation, such as osteolysis with subsequent implant failure [4,25,26] and chondromalacia in septic synovitis [6,27]. Additionally, persistent biofilms serve as a nidus for dissemination of infection to the bloodstream and vital organs, which can be life-threatening [2,28–31]. The complications related to biofilm infections are dire; 26–55% of people with orthopedic implant infections die within 5 years of initial diagnosis [29,31]. Persistent orthopedic biofilm infections are an equally significant problem in horses and are often caused by *Staphylococcus aureus* and *Escherichia coli*, which readily form biofilms on metallic orthopedic implants [7,32–34] and in synovial fluid as floating aggregates [12,35–38]. The resulting chronic lameness, loss of athletic potential, and reduced quality of life due to chronic, unresolved orthopedic infections [4,6,22,24] make euthanasia ultimately necessary in up to 54% of horses [4,5,22,39,40]. A treatment that disrupts established orthopedic biofilms and improves success of antimicrobials and surgical intervention is desperately needed.

Mesenchymal stromal cells (MSC) derived from equine bone marrow are immunomodulatory and used for the treatment of musculoskeletal injury and osteoarthritis in horses [41–47]. Recently, bone marrow-derived MSC from humans and horses have been demonstrated to secrete antimicrobial peptides *in vitro* [48–52], *ex vivo* [53]. Production of antimicrobial factors by MSC have also been demonstrated in murine and canine models of infection [54–56]. Equine bone marrow-derived MSC exert antimicrobial effects in a paracrine manner, via secretion of antimicrobial peptides. Cathelicidin is one such antimicrobial peptide that causes microbial growth inhibition bacterial cell membrane depolarization [49,51] and interferes with bacterial metabolism of key nutrients [50]. Equine bone marrow-derived MSC also enhance the host antibacterial response through stimulation of phagocytosis by neutrophils [52] and secretion of antimicrobial peptides from keratinocytes [53]. MSC from the adipose tissue of rats and mice or from human menses have been used successfully with antimicrobials to significantly reduce bacterial numbers [55–57]. TLR-activation of equine MSC used in combination with vancomycin successfully reduced bacterial counts in an equine model of multi-drug resistant *S*. *aureus* [52]. This concept of synergy between MSC and antimicrobials has high therapeutic potential.

To date, studies have primarily evaluated the ability of MSC to prevent establishment of infection, including planktonic (free-floating) bacterial reduction and inhibition of biofilm formation [49–52]. Harman et al. demonstrated that conditioned medium from equine bone marrow-derived MSC prevented biofilm formation by planktonic cultures of *S*. *aureus* and *E*. *coli* [49]. However, it was unclear if the anti-biofilm properties were due to bacterial killing prior to biofilm establishment, or direct disruption of biofilm formation. The biofilm matrix physically protects indwelling bacteria from being killed by immune cells and can utilize the host immune response to its advantage by incorporating secreted proteins [12,37,58] and DNA [59] into its matrix, thereby enhancing the barrier function of the biofilm. Therefore, it is important to investigate the ability of equine MSC to reduce established orthopedic biofilms containing a mature extracellular matrix. It is also unknown whether equine MSC can synergize with antimicrobials used clinically in equine orthopedic infections [21,60,61] to reduce established orthopedic biofilms to a greater extent than MSC alone.

The objectives of this study were to evaluate 1) the ability of equine bone marrow-derived MSC to reduce biomass (total cells and matrix), biofilm size, and live bacterial counts of established *S*. *aureus* or *E*. *coli* biofilms *in vitro*, and 2) whether the combination of MSC and the antimicrobial amikacin sulfate would be more effective than either alone. We hypothesized that 1) MSC would reduce biomass, size, and live bacterial counts of established *S*. *aureus* and *E*. *coli* biofilms compared to untreated controls, and 2) the combination of MSC with amikacin would reduce biomass, size, and live bacteria of *S*. *aureus* and *E*. *coli* biofilms more than MSC or amikacin alone.

## Materials and methods

### Overview

**Fig 1:**
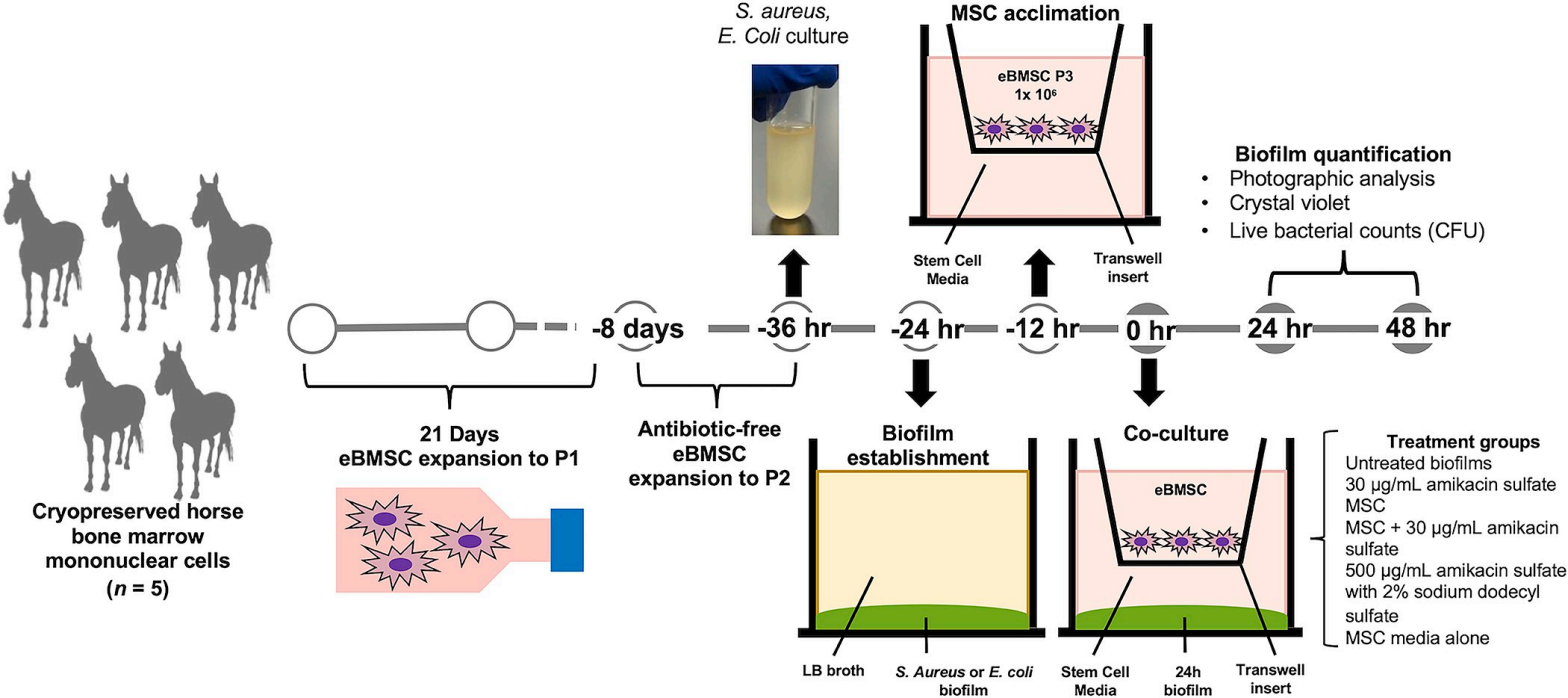
Experimental workflow to quantify biofilm reduction. MSC and biofilms of *S*. *aureus* or *E*. *coli* were prepared separately. Biofilms were established on 24-well tissue culture-treated plates for 24 hours and were co-cultured with 1 x 10^6^ passage 3 MSC seeded in transwell inserts alone or with amikacin sulfate for an additional 24 or 48 hours. Biofilms that remained untreated for the co-culture period were the negative control. Open circles indicate preparatory steps and solid circles indicate steps to establish co-cultures and perform biofilm quantification assays.

MSC were isolated from bone marrow from 5 horses and co-cultured at passage 3, alone or with a concentration of amikacin sulfate achieved *in vivo* [21,62], with established 24-hour *S*. *aureus* or *E*. *coli* biofilms in a transwell plate system (Fig 1). This system allowed MSC and biofilms to interact in a paracrine manner, without direct contact, during co-culture and facilitated easy biofilm separation after co-culture for imaging and quantification of biomass and live bacterial counts. Biofilm biomass, live bacterial counts, and biofilm size were quantified following 24 or 48 hours of co-culture.

### MSC isolation

Five healthy Thoroughbred horses (5.4 ± 1.5 years; 2 females and 3 male castrate) were placed under routine standing sedation and analgesia using detomidine hydrochloride (0.01–0.02 mg/kg IV). The sternum was clipped and aseptically prepared and the skin and subcutaneous tissues were anesthetized with 2% lidocaine. Using a Jamshidi needle, 60 mL bone marrow was aspirated into sodium heparinized syringes (200 units/mL marrow) from the 4^th^ or 5^th^ sternebrae [63–65]. Horses were administered flunixin meglumine (1.1 mg/kg IV) to provide analgesia immediately following bone marrow aspiration. All experimental procedures were approved by the Virginia Tech Institutional Animal Care and Use Committee (Protocol #20–136). Bone marrow mononuclear cells were isolated using gradient density centrifugation (Ficoll® Paque PLUS), assessed by live-dead staining with trypan blue (Gibco^TM^) and cryopreserved at passage 0 in fetal bovine serum (FBS) with 10% dimethyl sulfoxide until use [63–65]. Equine bone marrow mesenchymal stromal cells (MSC) were thawed in a 37°C water bath and plated at 350,000 live cells/cm^2^ in 175 cm^2^ tissue culture flasks in MSC medium (low-glucose GlutaMax DMEM with 110 μg/mL sodium pyruvate (Gibco^TM^)) with antibiotic (100 U/mL sodium penicillin and 100 μg/mL streptomycin sulfate (Sigma Aldrich^®^)) and 10% FBS (Gibco^TM^) [66–68]. A 50% medium exchange was performed every 48 hours and MSC were harvested at 70–80% confluence as passage 1 prior to short-term cryopreservation (Synth-a-freeze^TM^, Gibco^TM^) in liquid nitrogen, vapor phase.

### Biofilm growth

Quality control testing was performed on commercial strains of *S*. *aureus* (ATCC^®^ 29213) or *E*. *coli* (ATCC^®^ 25922) by broth microdilution (Sensititre^TM^, ThermoFisher^TM^) to ensure that the minimum inhibitory concentration (MIC) of each drug fell within the expected ranges as outlined in the Clinical Laboratory Standards Institute reported as 1–4 μg/mL for ATCC^®^ 29213 and 0.5–4 μg/mL for ATCC® 25922 (69). The MIC of amikacin when tested in our laboratory was ≤ 4 μg/mL for ATCC^®^ 29213 and ATCC® 25922, using a 4 μg/mL amikacin disk as the lowest tested concentration. Planktonic cultures of *S*. *aureus* or *E*. *coli* were established by isolation of pure colonies on tryptic soy agar (TSA) and inoculation of one colony of either bacterium in Luria-Bertani (LB) broth. Cultures were grown overnight at 37°C at 200 rpm on an orbital shaker (Fisherbrand^TM^ Incubating Mini-Shaker, Fisher Scientific) to reach the exponential growth phase and then were serially diluted in LB broth to a final optical density of 0.05 at 600 nm. Biofilms were established by inoculation of 2 x 10^5^ colony-forming units (CFU)/well in 24-well, tissue-culture treated plates and grown at 37°C, 5% CO_2_, and 95% humidity for 24 hours to allow adequate bacterial attachment and matrix production [14,34].

### Treatment groups

Six treatment groups were established in triplicate for 24 or 48 hours Table 1. Untreated biofilm (untreated/ negative control); biofilm + 30 μg/mL amikacin sulfate (Avet Pharmaceuticals, Inc^®^) [21,62,69]; biofilm + MSC; biofilm + MSC + 30 μg/mL amikacin sulfate; biofilm + 500 μg/mL amikacin sulfate with 2% sodium dodecyl sulfate (positive control) [70–72]. Wells containing no biofilm served as contamination controls. All treatment groups were cultured in antibiotic-free MSC medium with 5% FBS. Biofilm and co-culture treatment groups were established as described below.

**Table 1:** Treatment groups for Biofilm-MSC co-culture.

		Biofilm (*S*. *aureus* or *E*. *coli*)	MSC in transwell insert (1.0 x 10^6^)	Amikacin (30 μg/mL)	Other
Treatment Groups	**Untreated/ negative control** ^**a**^	✓			
	**30 μg/mL amikacin** ^**a**^	✓		✓	
	**MSC** ^**a**^	✓	✓		
	**MSC + 30 μg/mL amikacin** ^**a**^	✓	✓	✓	
	**Positive control** ^**a**^				500 μg/mL amikacin sulfate with 2% sodium dodecyl sulfate
	**Contamination control** ^**a**^				

^a^ All treatment groups were cultured in antibiotic-free MSC medium with 5% FBS. Groups were established in triplicate for 24 and 48 hour time-points.

### Biofilm-MSC co-culture

Thawed MSC were expanded to passage 2 in antibiotic-free MSC medium with 10% FBS and seeded at passage 3 into inserts of 24-well transwell plates (6.5 mm insert diameter, 0.4 μm pore diameter, polyester, Corning^®^) at 1.0 x 10^6^ MSC/insert in antibiotic-free MSC medium with 5% FBS. MSC dose was chosen based on previous bone marrow-derived MSC concentrations to give conditioned medium for activity against *S*. *aureus in vitro* [52], as well as extrapolation from clinical and experimental doses of bone marrow-derived MSC for equine intra-articular injection [73–76]. MSC were acclimated to the inserts overnight at 37°C, 5% CO_2_, and 95% humidity prior to transfer to biofilm co-culture to establish MSC-biofilm co-cultures at time = 0 hours. 24-hour-old *S*. *aureus* and *E*. *coli* biofilms were centrifuged at 1,400 x g at room temperature (RT) for 10 minutes to concentrate biomass in the well bottom so as to prevent inadvertent aspiration of detached cell clumps or planktonic bacteria. The overlying LB broth was then aspirated and antibiotic-free MSC medium with 5% FBS alone (negative control) ± 30 μg/mL amikacin sulfate. Inserts containing MSC transferred to wells containing biofilms. All groups had a total system fluid volume of 500 μL. The bacterial CFU:MSC ratios at time 0 were 500 *S*. *aureus* CFU:1 MSC and 1,000 *E*. *coli* CFU:1 MSC.

### Photographic analysis of biofilm size

Following 24 and 48 hours of co-culture, inserts containing MSC were aseptically removed with forceps for downstream assays. Biofilms were examined grossly and digitally photographed (iPhone XR, Apple, Inc) in a commercial ring LED photography booth with a black backdrop and lens set to an object distance of 19 cm (PULUZ^®^). High resolution JPEG images were imported to ImageJ (NIH) and converted to 8-bit greyscale. Binary images that showed biofilms as white central areas on a black background were obtained via default algorithm histogram thresholding (black value 0, white value 255) and the number of white pixels was recorded in an oval region of interest that incorporated the entire biofilm. Triplicate technical replicates were measured and the mean used for statistical analysis. Four blinded reviewers (K. Hackler, J. Field, H. Elshafie, S. Schumaker) were trained to review biofilm area using Image J. Each reviewer assessed images from all horses within a randomly assigned bacterial strain and time point. Reviewers were then randomly assigned to a different bacterial strain and time point for review so that each set of images was reviewed by two reviewers. Random allocation by time point and bacteria grouping was chosen as no direct comparisons between time point or bacteria were performed in final analysis. Pixel size was calibrated to reference lines of known distance in mm and final biofilm area in mm^2^ was calculated prior to statistical analysis.

### Biomass quantification

Following photography, total bacterial well contents were centrifuged at 1,400 x g for 10 minutes at RT, rinsed twice with distilled water and fixed with reagent grade methanol [77]. Well contents were stained with 0.2% aqueous crystal violet for 15 minutes at RT, excess stain aspirated, and unbound stain removed by rinsing once with distilled water. Biomass-bound stain was eluted with 70% ethanol for 15 minutes at RT, then 100 μL/well of eluted stain were transferred to a 96-well microtiter plate and optical density at 595 nm quantified on a spectrophotometric plate reader (BioTek® Synergy H1 ^TM^, Agilent Technologies, Inc) with commercial software (BioTek® Gen 5^TM^) [49,51,77].

### Live bacterial quantification

Total biofilm well contents were digested *in situ* with 20 μg/mL Proteinase K (Life Technologies, Inc) for 30 minutes at 37°C on a rotary shaker at 150 rpm [12]. Well digests were transferred to a sterile microfuge tube and wells rinsed 3 times with sterile phosphate-buffered saline (PBS) to collect residual bacteria. Digests were serially diluted 10-fold in PBS, plated in triplicate on TSA, and incubated overnight at 37°C. Live colonies were counted, divided by the dilution factor, and the quotient multiplied by well volume to calculate live bacteria/well system (CFU per biofilm):

$$CFU\;per\;Biofilm=\frac{Live\;Colonies}{Dilution\;Factor}\times Well\;Volume\;(mL)$$

### Statistical analysis

Sample size was calculated using commercial software (G*Power, University of Düsseldorf, Netherlands) based on *in vitro* data on mean antimicrobial peptide synthesis and bacterial killing by equine bone marrow-derived MSC [48,49] and using an effect size of 0.5, alpha level of 0.05, and power of 0.8. For biofilm area quantification, correlation between pixel measurements of the two independent investigators was assessed using Pearson’s r coefficient. Data distribution was assessed using normal probability plots, summarized as mean ± standard deviation (SD) if normally distributed, and effects of treatment on outcomes were analyzed using a mixed model analysis of variance. CFU results were log_10_-transformed to normalize distribution prior to analysis. The generalized linear model specified treatment, time, and the interaction between treatment and time as fixed effects. Horse identification was specified as a random effect. The interaction between treatment and time was further analyzed to compare the treatment groups at each time point. Where appropriate, p-values were adjusted for multiple comparisons using Tukey’s procedure. Statistical significance was set to p < 0.05. All analyses were performed using SAS version 9.4 (SAS Institute).

## Results

### MSC reduced biofilm organization and gross biofilm area

**Fig 2:**
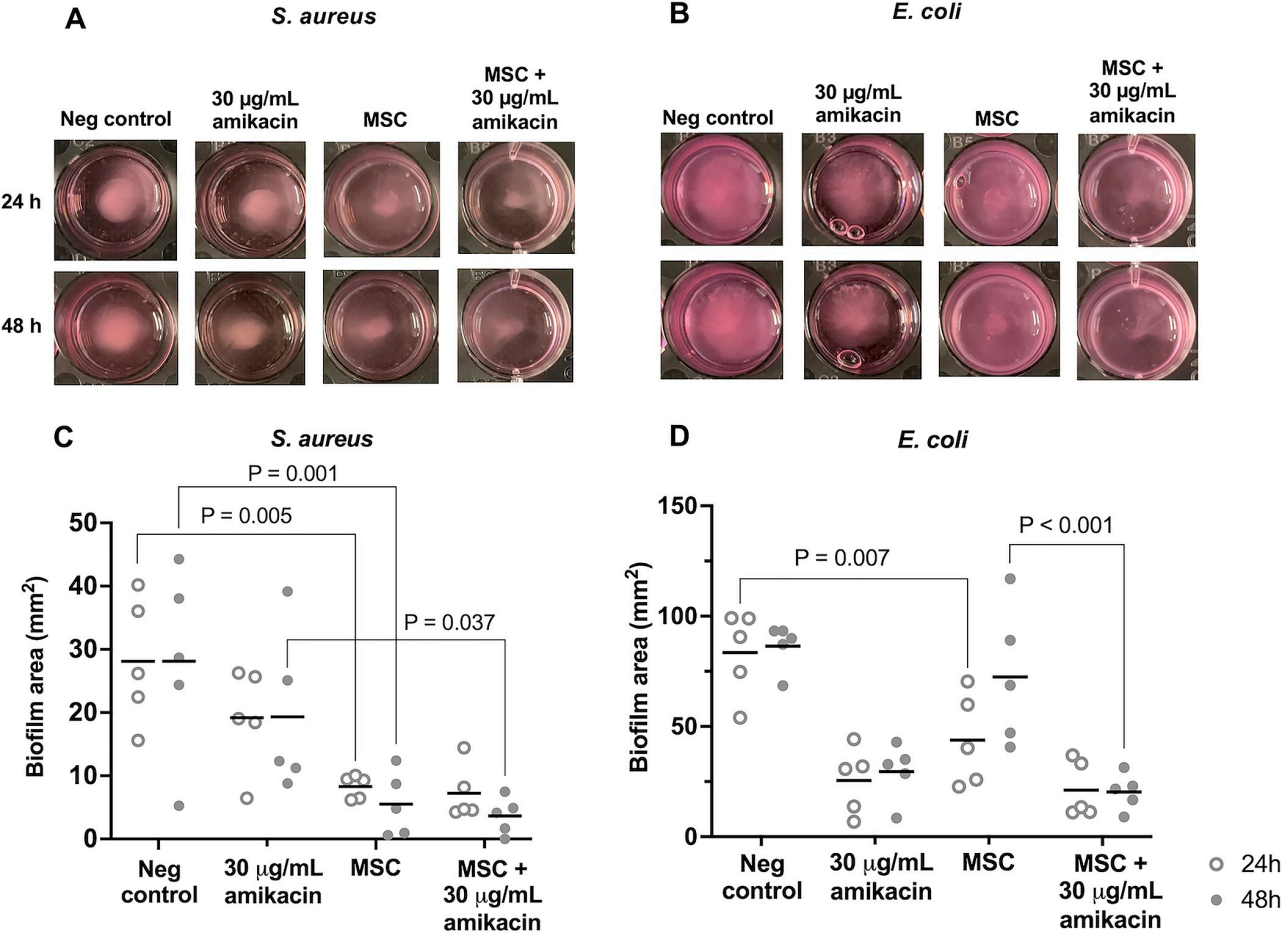
Effect of MSC ± amikacin on biofilm size. *In situ* digital images of biofilms from one representative horse for *S*. *aureus* (A) and *E*. *coli* (B) following 24 or 48 hours of treatment. The negative control was untreated biofilms. (C, D) Dot-plots of normalized biofilm area (mm^2^) from *n* = 5 horses. Horizontal black lines indicate mean biofilm area across horses, and circles indicate mean biofilm area for each individual horse at 24 (open circles) or 48 hours (closed circles) as measured from photographic analysis. Brackets and p-values indicate groups significantly different (p < 0.05) from each other.

MSC maintained 75% of baseline viability following co-culture with *S*. *aureus* biofilms and 70% following co-culture with *E*. *coli* biofilms for 48 hours. Biofilms were successfully established as a central aggregate that protruded up from the bottom of the well toward the meniscus. Established biofilms of each bacterial species that were then co-cultured with MSC were visibly less organized and had smaller, less-defined biofilms compared to untreated biofilms at both time points (Fig 2A and 2B). *E*. *coli* biofilms were less organized, with a more expansive central region, than *S*. *aureus* biofilms across treatment groups. When placed under mechanical stress on the same rotary shaker used for enzymatic biofilm digestion at 200 rpm, MSC-treated biofilms subjectively dispersed more readily than untreated or amikacin-treated biofilms (S1 and S2 Videos).

Correlation between observers for biofilm area using pixel thresholding was high, with a Pearson’s r coefficient of 0.911 (95% CI 0.865–0.940, p < 0.001). *S*. *aureus* biofilm area was significantly affected by treatment only (F = 12.42, df = 3, p < 0.001), whereas *E*. *coli* biofilm area was affected by treatment (F = 16.46, df = 3, p < 0.001) and time (F = 7.81, df = 3, p = 0.013), with a significant interaction between treatment and time (F = 4.65, df = 1, p = 0.016). MSC reduced *S*. *aureus* biofilm area compared to untreated biofilms at 24 (p = 0.005) and 48 hours (p = 0.001), while MSC + amikacin reduced *S*. *aureus* biofilm area compared to amikacin alone at 48 hours (p = 0.037), with no difference at 24 hours (p = 0.154) or compared to MSC alone at 48 hours (p = 0.998) (Fig 2C). MSC reduced *E*. *coli* biofilm area compared to untreated biofilms at 24 hours (p = 0.007) with no changes at 48 hours (p = 0.606) (Fig 2D). MSC + amikacin reduced *E*. *coli* biofilm area compared to MSC alone (p < 0.001) at 48 hours, with no changes at 24 hours (p = 0.202) or compared to amikacin at either timepoint (p = 0.980 at 24 hours; p = 0.843 at 48 hours).

### Co-culture with MSC reduced total biomass of *S*. *aureus* biofilms

**Fig 3:**
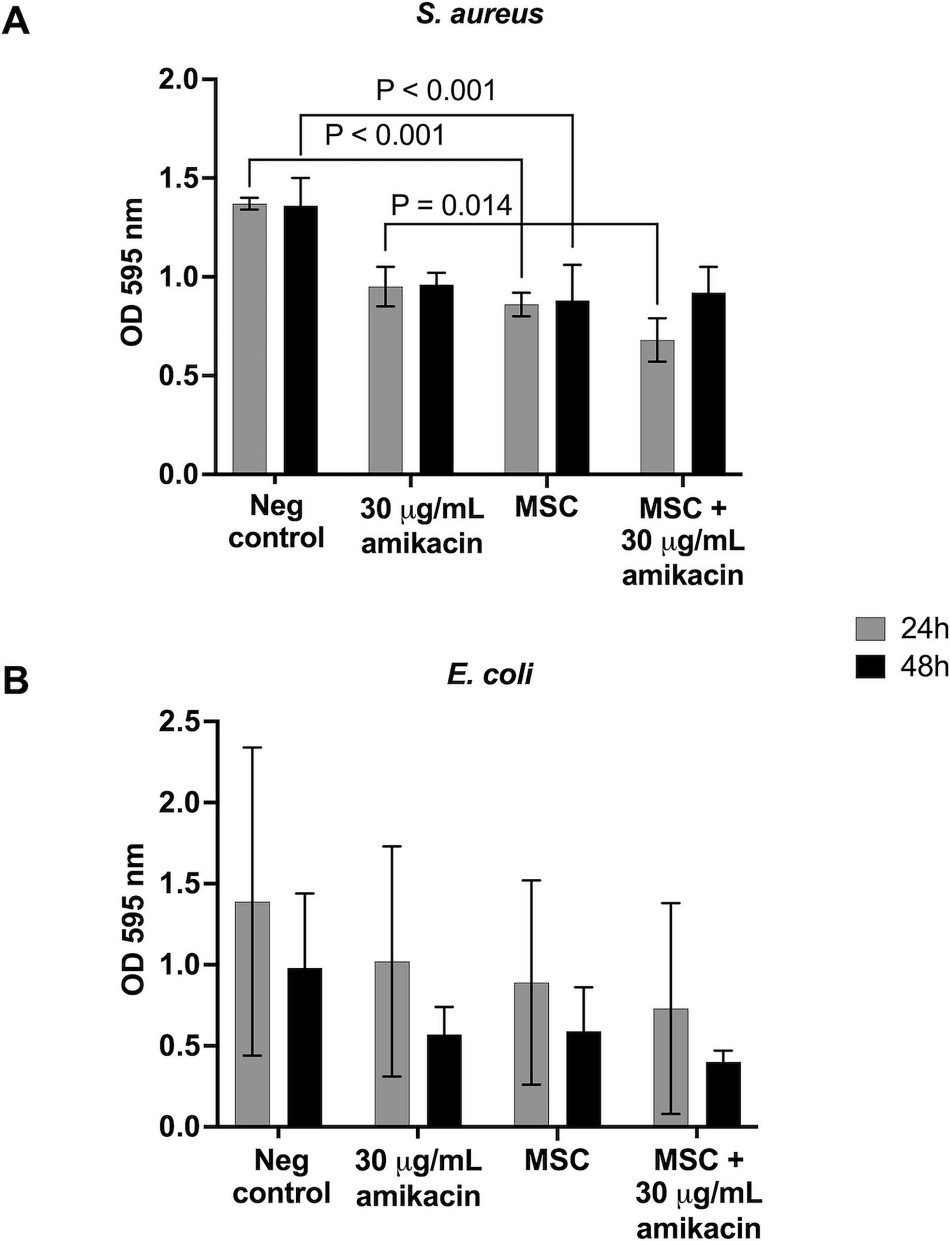
Biofilm biomass quantified by crystal violet staining for *S*. *aureus* and *E*. *coli*. Bars represent mean ± SD from biofilms co-cultured with MSC from *n* = 5 horses at 24 (grey) or 48 hours (black). The negative control was untreated biofilms. Brackets and p-values indicate groups significantly different from each other.

Co-culture with MSC reduced *S*. *aureus* biomass compared to untreated biofilms at 24 and 48 hours (p < 0.001 for both) (Fig 3A). MSC with amikacin reduced *S*. *aureus* biomass compared to amikacin alone at 24 (p = 0.014) and 48 hours (p = 0.053). MSC + amikacin reduced biomass of *E*. *coli* biofilms compared to untreated controls at 24 (p = 0.032) and 48 hours (p = 0.023) (Fig 3B). No other differences were detected between treatment groups.

### Co-culture with MSC reduced *S*. *aureus* CFU at 48 hours, with no improvement in bacterial killing of either species by MSC + amikacin versus amikacin alone

**Fig 4:**
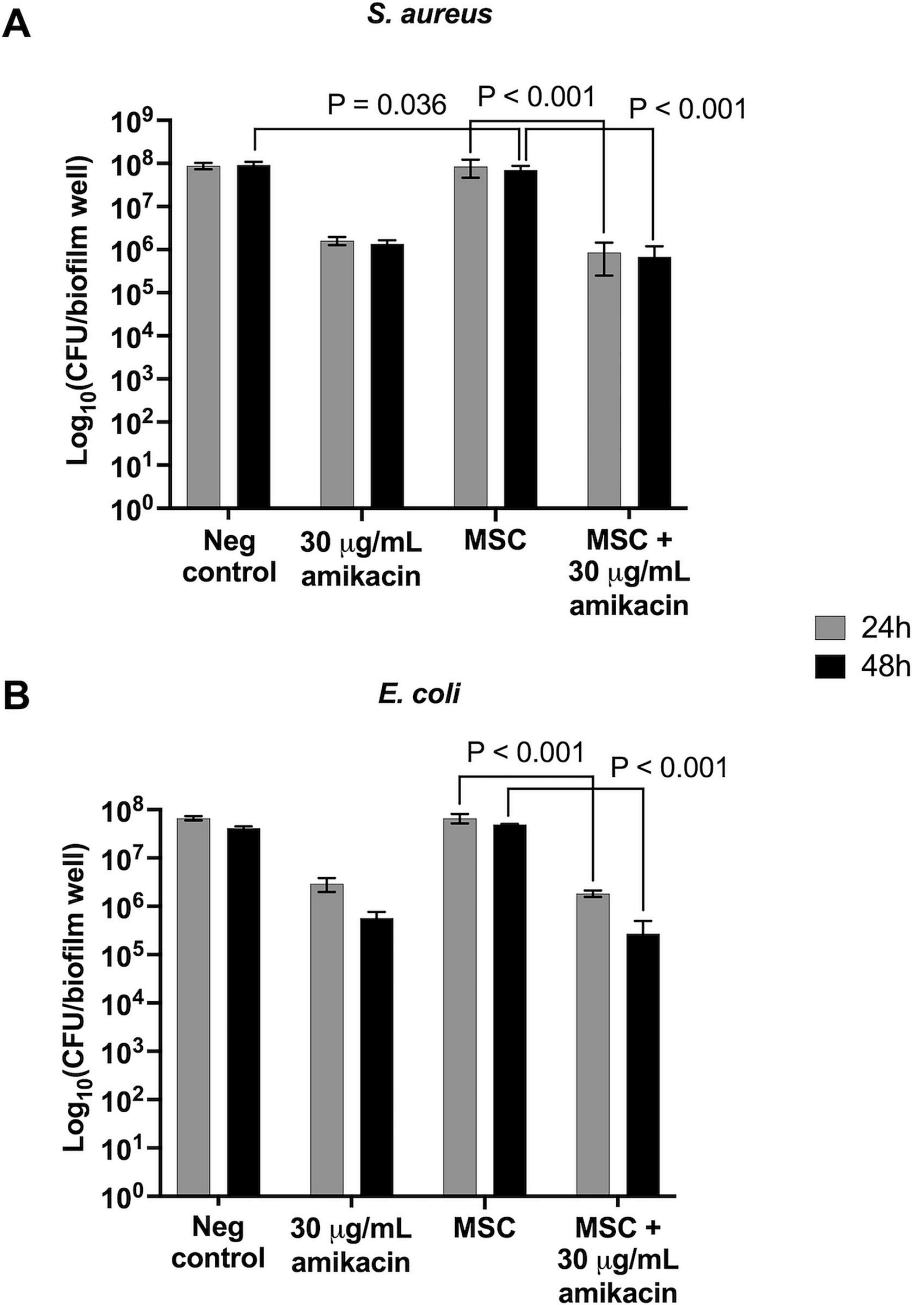
Live bacteria quantified by total colony forming units (CFU) per biofilm for *S*. *aureus* and *E*. *coli*. Bars represent mean ± SD from biofilms co-cultured with MSC from *n* = 5 horses at 24 or 48 hours. The negative control was untreated biofilms. Brackets indicate groups significantly different from each other.

CFU/biofilm of MSC-treated S. aureus biofilms was reduced by 0.5 log_10_-fold compared to untreated biofilms at 48 hours (p = 0.036), with no difference at 24 hours (p = 0.997) (Fig 4A). MSC + amikacin reduced CFU of *S*. *aureus* biofilms compared to MSC-treated biofilms by 2 log_10_-fold at both time points (p < 0.001). As for *S*. *aureus*, MSC + amikacin reduced *E*. *coli* CFU by 2 log_10_-fold compared to MSC alone at both timepoints (p < 0.001) (Fig 4B). However, reductions in CFU of both bacteria by MSC + amikacin were equivalent to reductions achieved by amikacin alone. Additionally, MSC + amikacin treatment reduced CFU of both bacteria by 2 log_10_-fold at both timepoints compared to untreated controls (p < 0.001). No other differences were detected between treatment groups.

## Discussion

Our study demonstrates that equine bone marrow-derived MSC reduce biomass and the biofilm area of established *S*. *aureus* biofilms, but that MSC + amikacin did not reduce biomass further compared to MSC alone. However, MSC + amikacin was effective at reducing *S*. *aureus* biofilm biomass compared to amikacin alone at 24 hours and at reducing biofilm area compared to amikacin alone at 48 hours. Taken together, our results are supportive of a potential synergy between amikacin and MSC in combatting *S*. *aureus* biofilms. In contrast, while *E*. *coli* biofilm area and visible biofilm organization were reduced by MSC at 24 hours and by MSC + amikacin at 48 hours compared to no treatment, neither MSC nor MSC + amikacin decreased *E*. *coli* biofilm biomass. With the exception of *S*. *aureus* following 48 hours of MSC treatment, MSC did not otherwise reduce live bacterial counts in our biofilms. Furthermore, MSC + amikacin reduced live counts of both bacteria equivalently to amikacin alone.

MSC response is paracrine in nature based on co-culture of planktonic bacteria with either equine bone-marrow derived MSC or MSC-conditioned medium [49–51]. Our transwell system was chosen over using conditioned medium to enable a MSC-mediated antibacterial response through real-time paracrine feedback without allowing physical contact with the bacteria. MSC from horses and humans express pattern recognition receptors (PRR) [52,78–80] that can be stimulated by bacterial pathogen-associated molecular patterns or synthetic ligands to enhance planktonic bacterial killing via antimicrobial peptide synthesis [48,50,52]. Our co-culture model will enable future investigations using pre-stimulated MSC. A survey of equine veterinary practitioners found that 72.1% use bone marrow-derived MSC at least once a year for orthopedic conditions [81]. Therefore, use of MSC versus conditioned medium also more closely aligns with current clinical practice of MSC injection and more closely aligns with the use of horses as a naturally occurring model in future studies.

Although our co-culture design allowed for stimulation of MSC PRR stimulation by bacterial products, it is unclear why a more uniform anti-biofilm MSC response was not observed. The *S*. *aureus* and *E*. *coli* strains used in our study were selected because of their ability to form robust biofilms, their clinical relevance [82,83], and their susceptibility to amikacin sulfate [84]. However, evaluation of biofilm-forming strains that generate a strong inflammatory response, such as methicillin-resistant *USA300* [25,51,53], may stimulate a greater anti-biofilm MSC response than that seen in our study. Pre-treatment of MSC to increase their immunomodulatory properties prior to bacterial co-culture may also improve biofilm reduction. Possible candidates include PRR agonists [50,52,85], hypoxic exposure [85], or three-dimensional culture [86,87]. Inclusion of neutrophils in this model may have improved biofilm reduction and more closely captured the MSC antibiofilm potential that would occur *in vivo* [52,55,73]. MSC may indirectly exert an antimicrobial effects through enhancement of the antibacterial responses of immune cells, such as macrophages and neutrophils, present in infected tissues [55,88].

Our study demonstrated a difference in the ability of MSC to combat established biofilms of *S*. *aureus* versus *E*. *coli*. These findings are consistent with greater reduction of live bacterial counts for Gram-positive versus Gram-negative organisms by equine adipose-derived MSC [89] and platelet lysate [35]. There may be differences in the response of MSC to *S*. *aureus* versus *E*. *coli* biofilms related to differences in biofilm matrix composition as *S*. *aureus* and *E*. *coli* biofilms differ in their specific matrix carbohydrates [11,90] and adhesion proteins [8,10,91]. Activation of the MSC antimicrobial response is driven by PRR which may differ between biofilms versus planktonic bacteria, or between bacterial species. Activation of equine MSC with a TLR3 agonist did not increase the anti-biofilm effect against *S*. *aureus*, despite successfully reducing planktonic *S*. *aureus in vitro* [52]. When equine allogeneic MSC were pre-stimulated with the same TLR3 agonist and used in an equine model of multi-drug resistant *S*. *aureus* synovial infection, bacterial numbers increased at 24 hours, but then were significantly reduced by 96 hours [73]. These findings indicate that a longer incubation time may be needed to demonstrate anti-biofilm effects, or optimization using specific or combination PRR activation is needed. The importance of specific PRR activation has been demonstrated using human umbilical cord blood derived MSC that had enhanced activity against *E*. *coli* via the TLR4 pathway but not the TLR2 pathway [92].

Donor-dependent variation in MSC anti-biofilm activities was observed with both bacterial strains but was more pronounced for *E*. *coli* and may have impacted our ability to detect differences between treatment groups. In particular, there was greater variation in *E*. *coli* untreated (negative control) and amikacin-treated biofilm biomass. Bone marrow-derived MSC from different horses vary in their immunomodulatory properties [86,93], proliferation [94], differentiation [94], and cell yield on isolation from tissues [95]. The potential for inter-donor variation in overall MSC anti-biofilm function and in the ability of *E*. *coli* versus *S*. *aureus* to stimulate anti-biofilm responses requires further investigation. Donor screening [96,97] or pooling of active factors from multiple horses [35,36] may be required to minimize the impact of inter-donor variation.

The primary mechanism of MSC against established *S*. *aureus* and *E*. *coli* biofilms in this study was dispersion of the biofilm matrix based on observed reductions in *S*. *aureus* and *E*. *coli* biofilm size and *S*. *aureus* biofilm matrix components. Equine bone marrow-derived MSC kill planktonic bacteria and prevent biofilm formation by disrupting bacterial cell membranes through secretion of amphipathic antimicrobial peptides [48,49] and cysteine proteases [51]. MSC antimicrobial proteins may disrupt the matrix of established biofilms in a similar fashion, causing reductions in biomass and biofilm area.

With the exception of *S*. *aureus* following a 48 hour co-culture, MSC did not reduce live bacteria of our biofilms compared to untreated biofilms. The reasons for this lack of correlation between live bacterial numbers and reduced biomass and biofilm area are uncertain. Biofilm area measured only the biofilm whereas CFU measures the sum of viable bacterial from both the biofilm and the suspension. The lack of reduction in CFU after biofilm reduction indicates that either (a) the bacteria removed from the biofilm remained viable or (b) the surviving bacteria that were removed were able to reproduce more quickly in the suspension than in the biofilm. However, ongoing multiplication of dispersed bacteria prior to quantification may have masked these immediate reductions, as planktonic bacteria divide more rapidly than biofilm-bound bacteria [16]. Three-dimensional imaging techniques, including confocal microscopy [12] or scanning electron microscopy [26], may have captured MSC-specific biofilm reductions not seen with our techniques. While MSC-treated biofilms were subjectively easier to disperse under mechanical stress in our study, biomechanical testing could be used to quantify changes in biofilm matrix physical properties, such as stiffness [14], following MSC treatment. The potential for MSC to disperse biofilm matrix may prove useful in a therapeutic context by increasing antimicrobial and immune cell access to live bacteria in biofilms, resulting in improved infection clearance. Biofilm matrix dispersion by MSC may also facilitate physical removal of biofilms intraoperatively by pulsatile lavage or ultrasonic debridement [98]. Staged treatment of biofilms with MSC to initially disperse the matrix, followed by surgical debridement to remove residual matrix and antimicrobials to kill liberated live bacteria, may be a useful approach to improve the therapeutic utility of biofilm matrix dispersal.

Use of established biofilms with a mature matrix likely limited the ability of MSC and/or amikacin to penetrate the biofilm matrix compared to planktonic bacteria. Our MSC:bacteria ratio or the physiologic dose of amikacin sulfate chosen, may have resulted in lower than expected live bacterial reductions [21,62]. Ratios of MSC to live bacteria used in this study were similar to those reported for a 6-hour co-incubation of human MSC with planktonic *S*. *aureus* or *E*. *coli* in a transwell plate system [48] and were chosen to maximize the MSC dose while maintaining MSC survival during co-culture. The concentration of amikacin used in our study was chosen as the minimum concentration to give a C_max_:MIC ratio of 8:1 for our *S*. *aureus* and *E*. *coli* strains (MIC_90_ ≤ 4 μg/mL) [21,99–101], as has been historically described to maximize the antibiotic effect of amikacin. Amikacin is rapidly cytotoxic to equine bone marrow-derived MSC in a concentration dependent manner [102]. The 30 μg/mL dose selected reflected a concentration in the middle of the non-cytotoxic range for 24 hours of exposure and maintained MSC viability after 48 hours in our study conditions [103]. Concentrations of amikacin in synovial fluid vary widely following regional limb perfusion in horses [104]. The selected concentration is within the expected range of concentration and fold-MIC for our isolates [21,62,69,104]. However, because the presence of a biofilm matrix increases the MIC of antimicrobials 100–2,000 times [12,15] compared to planktonic bacteria, our amikacin concentration may have been insufficient in the face of established biofilms. Further elucidation of synergy between amikacin and MSC will require testing varying doses of amikacin and MSC.

Amikacin was preferentially selected over other aminoglycosides or antibiotic classes as it is commonly used during regional limb perfusion in horses [104] and 78.7% of veterinarians who use antimicrobials during intra-articular injection use amikacin [105]. Adaptive resistance to aminoglycoside antimicrobials *in vitro* can develop within 24 hours of constant antimicrobial exposure [106,107] and could have limited live bacterial count reductions in groups treated with amikacin and our ability to detect synergy between MSC and amikacin. Antimicrobial peptides secreted by equine bone marrow-derived MSC inhibit the growth of bacteria commonly found in skin wounds [49,51] but their effect on antimicrobial resistant microbes remains unknown. Antimicrobial resistant infections arise in clinical patients for reasons including chronic infections containing mature biofilms [6,108], following prolonged antimicrobial pre-treatment in which adaptive resistance develops [27], or polymicrobial infections [22,28,109]. Areas that require future investigation include the ability of MSC to disrupt biofilms with varying degrees of potential treatment resistance, testing a range of amikacin concentrations above fold-MIC and using other antimicrobial classes in conjunction with MSC.

## Conclusions

Our study documents MSC-mediated reductions in biomass for *S*. *aureus* biofilms and biofilm area for *S*. *aureus* and *E*. *coli* biofilms. The transwell co-culture model facilitated culture of MSC and biofilms within the same fluid space while allowing physical separation to enable downstream assays for established biofilms. We demonstrated that the primary effect of MSC for established biofilms of *S*. *aureus* and *E*. *coli* was reduction of biofilm matrix. The dose and conditions of MSC tested did not show an appreciable effect on live bacterial counts of either *S*. *aureus* or *E*. *coli*. The lower-than-expected live bacterial reduction by MSC does not correlate with biofilm matrix reduction and may reflect limitations in quantification techniques, culture conditions, or dispersal of live bacteria that were not subsequently killed by MSC. MSC affected *E*. *coli* biofilms to a lesser extent than *S*. *aureus* biofilms, which warrants further investigation into differences in resistance of *E*. *coli* biofilm matrix to MSC-mediated disruption, or in stimulation of an MSC anti-biofilm response. Our results support further investigation of the mechanisms by which MSC disrupt biofilm matrix and highlights the importance of targeting specific bacterial strains for optimization of MSC anti-biofilm therapy.

## Supporting information

S1 Video *S*. *aureus* biofilms on a rotary shaker. S1 Video of *S*. *aureus* biofilms on a rotary shaker (Fisherbrand^TM^ Incubating Mini-Shaker) at 200 rpm after 48 hours of treatment demonstrates subjective biofilm dispersion under mechanical stress. Treatment groups in plate columns from left to right are: untreated biofilms (negative control), biofilms treated with 30 μg/mL amikacin sulfate, biofilms treated with MSC, or biofilms treated with MSC + 30 μg/mL amikacin sulfate. Similar relative biofilm dispersion between treatment groups was seen after 24 hours of co-culture.
(MOV)

S2 Video *E*. *coli* biofilms on a rotary shaker. S2 Video of *E*. *coli* biofilms on a rotary shaker (Fisherbrand^TM^ Incubating Mini-Shaker) at 200 rpm after 48 hours of treatment demonstrate subjective biofilm dispersion under mechanical stress. Treatment groups in plate columns from left to right are: untreated biofilms (negative control), biofilms treated with 30 μg/mL amikacin sulfate, biofilms treated with MSC, or biofilms treated with MSC + 30 μg/mL amikacin sulfate. Similar relative biofilm dispersion between treatment groups was seen after 24 hours of co-culture.
(MOV)
